# Relation Between Coronary Tortuosity and Vasomotor Dysfunction in Patients Without Obstructed Coronaries?

**DOI:** 10.3389/fcvm.2021.804731

**Published:** 2022-01-13

**Authors:** Tijn P. J. Jansen, Kyra van Keeken, Regina E. Konst, Aukelien Dimitriu-Leen, Angela H. E. M. Maas, Niels van Royen, Peter Damman, Suzette Elias-Smale

**Affiliations:** Radboudumc, Department of Cardiology, Nijmegen, Netherlands

**Keywords:** coronary tortuosity, coronary function test, microvascular dysfunction, vasospasm, IMR, CFR

## Abstract

**Background:** A large proportion of patients with angina and no obstructive coronary artery disease (ANOCA) has underlying coronary vasomotor dysfunction (CVDys), which can be diagnosed by a coronary function test (CFT). Coronary tortuosity is a common angiographic finding during the CFT. Yet, no data exist on the association between vasomotor dysfunction and coronary tortuosity.

**Aim:** To investigate the association between CVDys and coronary tortuosity in patients with ANOCA

**Methods:** All consecutive ANOCA patients who underwent clinically indicated CFT between February 2019 and November 2020 were included. CFT included acetylcholine spasm testing to diagnose epicardial or microvascular spasm, and adenosine testing to diagnose microvascular dysfunction (MVD). MVD was defined as an index of microvascular resistance (IMR) ≥ 25 and/or coronary flow reserve (CFR) <2.0. Coronary tortuosity, was scored (no, mild, moderate or severe) based on the angles of the curvatures in the left anterior descending (LAD) artery on angiography.

**Results:** In total, 228 patients were included (86% female, mean age 56 ± 9 years). We found coronary artery spasm in 81% of patients and MVD in 45% of patients (15%: abnormal CFR, 30%: abnormal IMR). There were 73 patients with no tortuosity, 114 with mild tortuosity, 41 with moderate tortuosity, and no patients with severe tortuosity. No differences were found in cardiovascular risk factors or medical history, and the prevalence of CVDys did not differ between the no tortuosity, mild tortuosity and moderate tortuosity group (82, 82, and 85%, respectively).

**Conclusion:** In this study, CVDys was not associated with coronary tortuosity. Future experimental and clinical studies on the complex interplay between coronary tortuosity, wall shear stress, endothelial dysfunction and coronary flow are warranted.

## Introduction

Worldwide, approximately 112 million people are affected by angina pectoris, which is the most common symptom of ischemic heart disease (IHD) (1). Often, angina pectoris is a consequence of a flow limiting stenosis of the coronary artery due to atherosclerosis. However, up to 50% of the patients undergoing coronary angiography because of angina are found to have no obstruction (2). These patients are referred to as “Angina with No Obstructive Coronary Artery Disease” (ANOCA). Previous studies have demonstrated that a large proportion of these ANOCA patients have underlying coronary vasomotor dysfunction (CVDys). This includes the endotypes epicardial vasospasm, microvascular vasospasm and/or microvascular dysfunction (MVD), the latter encompassing reduced coronary flow reserve (CFR) or increased microvascular resistance (3, 4). The gold standard to diagnose CVDys is invasive coronary function testing (CFT) (5), including coronary spasm provocation with acetylcholine and the assessment of microvascular function indices using adenosine.

Coronary tortuosity is a common angiographic finding and frequently observed in patients undergoing a CFT. It influences wall shear stress as bending of the vessel leads to disruption of laminar flow, resulting in high energy loss and increased shear stress in the outside bend on the vessel wall (6, 7). Coronary artery spasm is thought to be associated with wall shear stress through its relation with endothelial dysfunction, although studies show conflicting results as to whether it is a low or a high wall shear stress that is associated with endothelial dysfunction (8–10).

It has been proposed that in severe tortuosity, including kinking or coiling, flow alterations may lead to reduction of coronary perfusion pressure, eventually causing ischemia and thus anginal symptoms (6). These flow alterations and decrease in coronary perfusion might be detected by the assessment of coronary flow and microvascular function during the CFT. Recently, in a small pilot study of 8 patients with severe tortuosity, it was shown that mean coronary flow reserve was decreased and the index of microvascular resistance was increased (11).

However, to date, the relation between coronary tortuosity and the various endotypes of CVDys, as assessed by CFT, has not been evaluated. Therefore, the aim of our study was to evaluate the association of coronary tortuosity with the CVDys, including coronary spasm and MVD in patients with ANOCA.

## Methods

### Study Population

In this single-center, prospective study, we analyzed coronary angiographic images for tortuosity in 228 ANOCA patients that underwent a clinically indicated CFT between February 2019 and November 2020 at the Radboud University Medical Centre in Nijmegen, the Netherlands. All patients gave written informed consent.

### Clinical Characteristics

Clinical characteristics, including medical history, cardiovascular risk factors and symptom characteristics, were obtained from both the electronic patient file and an online patient questionnaire. This included questions regarding angina characteristics (12).

### Coronary Function Test

In patients clinically suspected of CVDys, first, a diagnostic coronary angiography was performed to rule out obstructive CAD defined as one or more epicardial stenoses of ≥50% or a fractional flow reserve (FFR) <0.8. When ruled out, the CFT was performed according to a standardized protocol, as has been described previously by Konst et al. (4) In short, ascending doses of 2, 20, 100, and 200 μg acetylcholine (ACH) were administered in the left coronary artery (LCA) through a guiding catheter with continuous monitoring of symptoms and ischemic ECG changes. Epicardial spasm was defined as vasoconstriction with a diameter reduction of ≥90%, combined with recognizable symptoms and ischemic ECG changes (12, 13). Focal epicardial spasm was diagnosed when epicardial spasm was observed within the boundaries of one isolated coronary segment, and diffuse epicardial spasm when >2 adjacent coronary segments showed epicardial spasm (12, 13). Microvascular spasm was diagnosed in the case of <90% coronary vasoconstriction, with recognizable symptoms and ischemic ECG changes (12). After administration of nitroglycerin to counteract coronary artery spasm, indices of MVD, including the coronary flow reserve (CFR) and the index of microvascular resistance (IMR), were measured using the thermodilution method adenosine (ADE) infusion to acquire maximal hyperaemia. Measurements were performed with the pressure/temperature wire placed in the distal 1/3 part of the LAD, independent of possible tortuous segments (14). The CFR is the ratio of hyperaemic-to-resting coronary flow velocity, which provides important information about the functional aspect of coronary circulation (12). The IMR, a measure of the microcirculatory resistance, represents structural microvascular abnormalities (12). MVD was defined as a CFR of <2.0 or/and an IMR of ≥25, in correspondence with current international consensus documents (1, 15).

### Tortuosity Measurements

**Figure 1:**
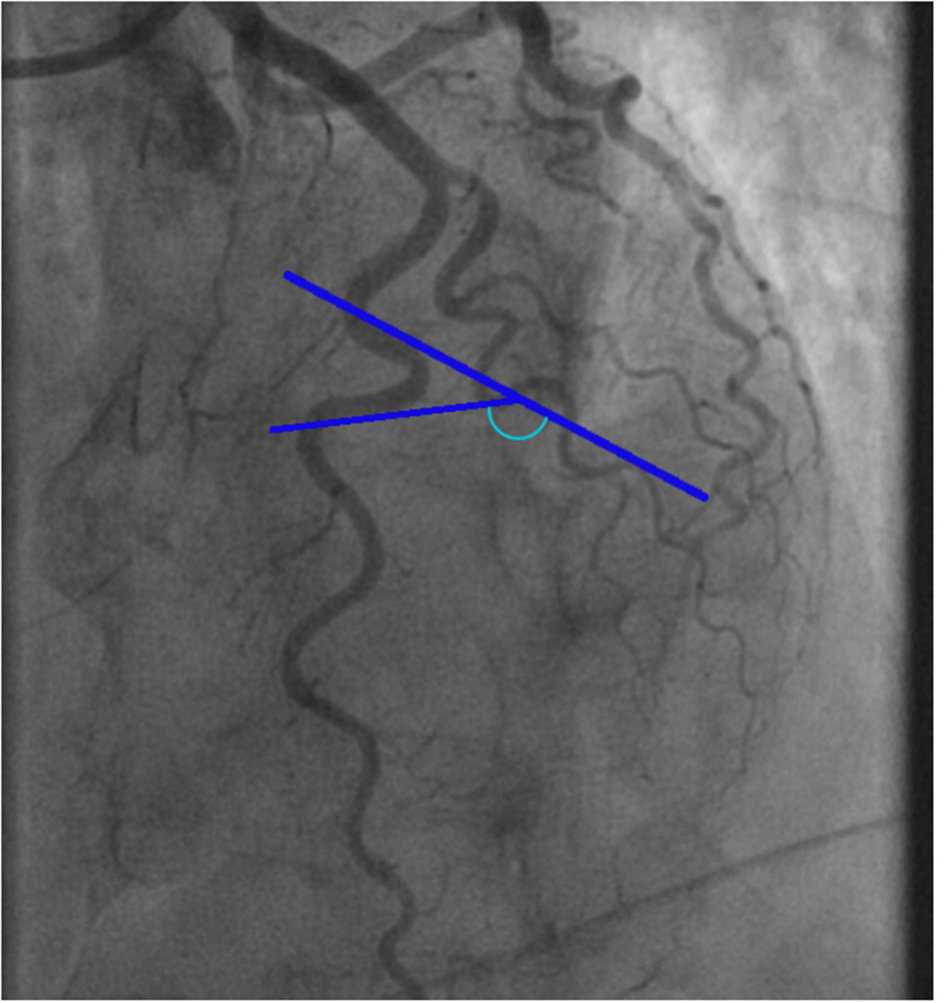
Example of a tortuosity measurement of the left anterior descending artery. The blue marked angle defines the angle of the curvature surrounded by blue lines.

**Figure 2:**
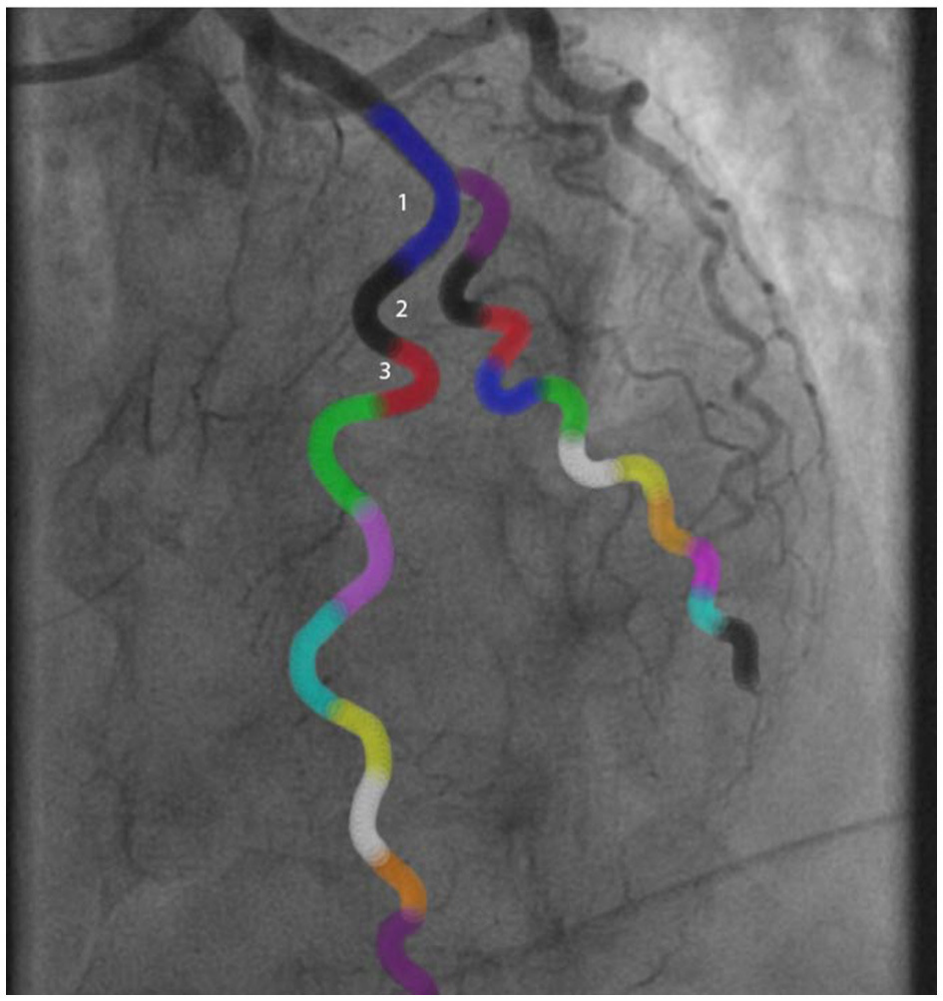
Example of a tortuosity assessment of the left anterior descending artery. The angles of all colored curvatures were measured to assess tortuosity: ^1^Blue curvature = 100°, ^2^Black curvature = 125°, ^3^Red curvature = 150°. In this case there was moderate tortuosity.

Since physiologic measurements during CFT were performed in the LAD artery, we also evaluated coronary tortuosity in the LAD artery. For the measurement of tortuosity, the left anterior oblique (LAO) 45-cranial 30° and the right anterior oblique (RAO) 30-cranial 30° views were used. The view after nitroglycerin infusion was primarily used, which was usually the LAO 45-cranial 30° view. Measurements of the other views were applied to estimate the angles of curvatures that were not entirely visualized on the LAO view. Measurements were performed at end-diastole as visualized in Figure 1. To quantify the severity of coronary tortuosity, the classification set up by Eleid et al. (7) was used. Mild tortuosity was defined as the presence of ≥3 consecutive curvatures of 45– 90° in the LAD artery or ≥3 consecutive curvatures of 90–180° in a diagonal of the LAD artery. Moderate tortuosity was defined as the presence of ≥3 consecutive curvatures of 90–180° in the LAD artery, and severe tortuosity was defined as the presence of ≥2 consecutive curvatures of ≥180° in the LAD artery. Patients who did not meet any of these criteria were defined as no tortuosity. The LAD artery were also examined on several additional markers of tortuosity (Table 1), including the S-curve sign, the intravessel symmetry sign, and the corkscrew sign, as described in the study by Ciurică et al. (16) Additionally, we considered the number of curvatures in the LAD artery as another marker of tortuosity (Figure 2).

**Table 1:** Markers of tortuosity: Definitions.

**Markers of tortuosity**	**Description**	**Examples**
S-curve sign	Curvatures on a coronary artery that are shaped like an S	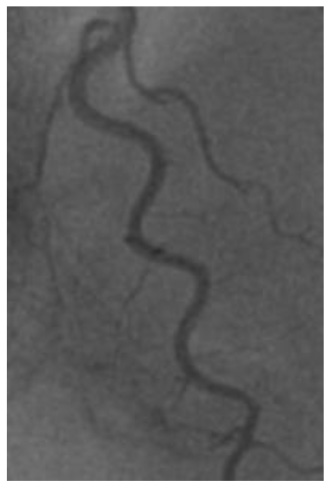
Intravessel symmetry sign	Symmetrical curvatures on a coronary artery	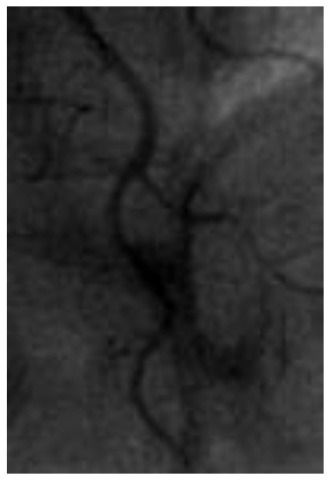
Corkscrew sign	Helical course of a coronary artery ≥360° perpendicular to the epicardial plane	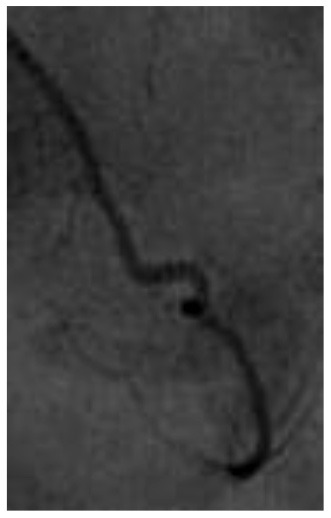

### Statistical Analyses

Continuous data are presented as mean ± standard deviation (SD), or median and interquartile interval, where appropriate. We used the Kolmogorov-Smirnov test to check for normal distribution of data. Categorical data are presented as numbers (%). Because none of the patients of our cohort had severe tortuosity, comparisons were made between three groups: no tortuosity, mild tortuosity, and moderate tortuosity. Differences between two groups were assessed by an independent sample *t*-test for continuous data with a normal distribution. Otherwise, the nonparametric Mann-Whitney U test was used. Differences between the three groups were assessed by one-way ANOVA or the nonparametric Kruskal-Wallis test, where appropriate. Categorical data were compared with the use of Fisher's exact test. Correction for possible confounders (i.e., variables that were associated with tortuosity *p* ≤ 0.10) was done using multinomial logistics. Correlations were assessed using the nonparametric Pearson correlation test. A two-sided *P* < 0.05 was considered statistically significant. In case of pairwise comparisons between three groups, Bonferroni correction was applied. In such cases, a *p* < 0.017 (=0.05/3) was considered statistically significant. All analyses were performed using SPSS Statistics version 25 (SPSS Inc., Chicago, IL, USA).

## Results

### Patient Characteristics

In total, 228 patients with ANOCA who underwent CFT were included in the analyses. All patients completed acetylcholine spasm testing, 14 patients did not undergo adenosine testing, mainly due to contra-indications (e.g., severe asthma). The mean age of all patients was 56 ± 9 years, and most patients were women (86%).

According to our method of tortuosity measurements, we found 73 patients (32%) with no tortuosity, 114 (50%) with mild tortuosity, 41 (18%) with moderate tortuosity and no patients with severe tortuosity.

Relevant medical history and cardiovascular risk factors showed no difference between the groups, as displayed in Table 2, although hypertension seemed to be more prevalent in increasing severity of tortuosity. Women had a higher prevalence of moderate tortuosity than men (20 vs. 3%, *p* = 0.029). The tortuosity groups did not differ in angina characteristics or medication use.

**Table 2:** Patient characteristics stratified for patients with no/mild/moderate tortuosity.

	**Total** ***N* = 228**	**No tortuosity** ***n* = 73**	**Mild tortuosity** ***n* = 114**	**Moderate tortuosity** ***n* = 41**	***p*-value**
Age, yrs	58 ± 8	57 ± 9	55 ± 8	57 ± 8	NS
Female	199 (87%)	58 (80%)	101 (88%)	40 (98%)	0.02
**Relevant medical history**				
History of ACS	39 (17%)	13 (18%)	19 (17%)	7 (18%)	NS
History of PCI	36 (16%)	16 (22%)	16 (14%)	4 (10%)	NS
**Cardiovascular risk factors**				
Hypertension	100 (44%)	27 (37%)	49 (43%)	24 (59%)	0.08
≥ 3 cardiovascular risk factors^*^	116 (51%)	37 (51%)	63 (56%)	16 (42%)	NS
**Angina characteristics**	*n* = 183	*n* = 57	*n* = 97	*n* = 29	
Symptoms at rest	159 (87%)	52 (91%)	82 (85%)	25 (86%)	NS
Symptoms during exercise	141 (62%)	46 (81%)	71 (74%)	24 (83%)	NS
Symptoms exerted by emotion or stress	121 (53%)	32 (56%)	69 (72%)	20 (69%)	NS

* *Cardiovascular risk factors include: adipose, hypertension, dyslipidaemia, diabetes, current/former smoker and premature coronary artery disease in first-degree relative*.

*Values are mean ± SD or %. NS, not significant*.

*ACS, acute coronary syndrome; PCI, percutaneous coronary intervention*.

### Coronary Spasm and Tortuosity

**Figure 3:**
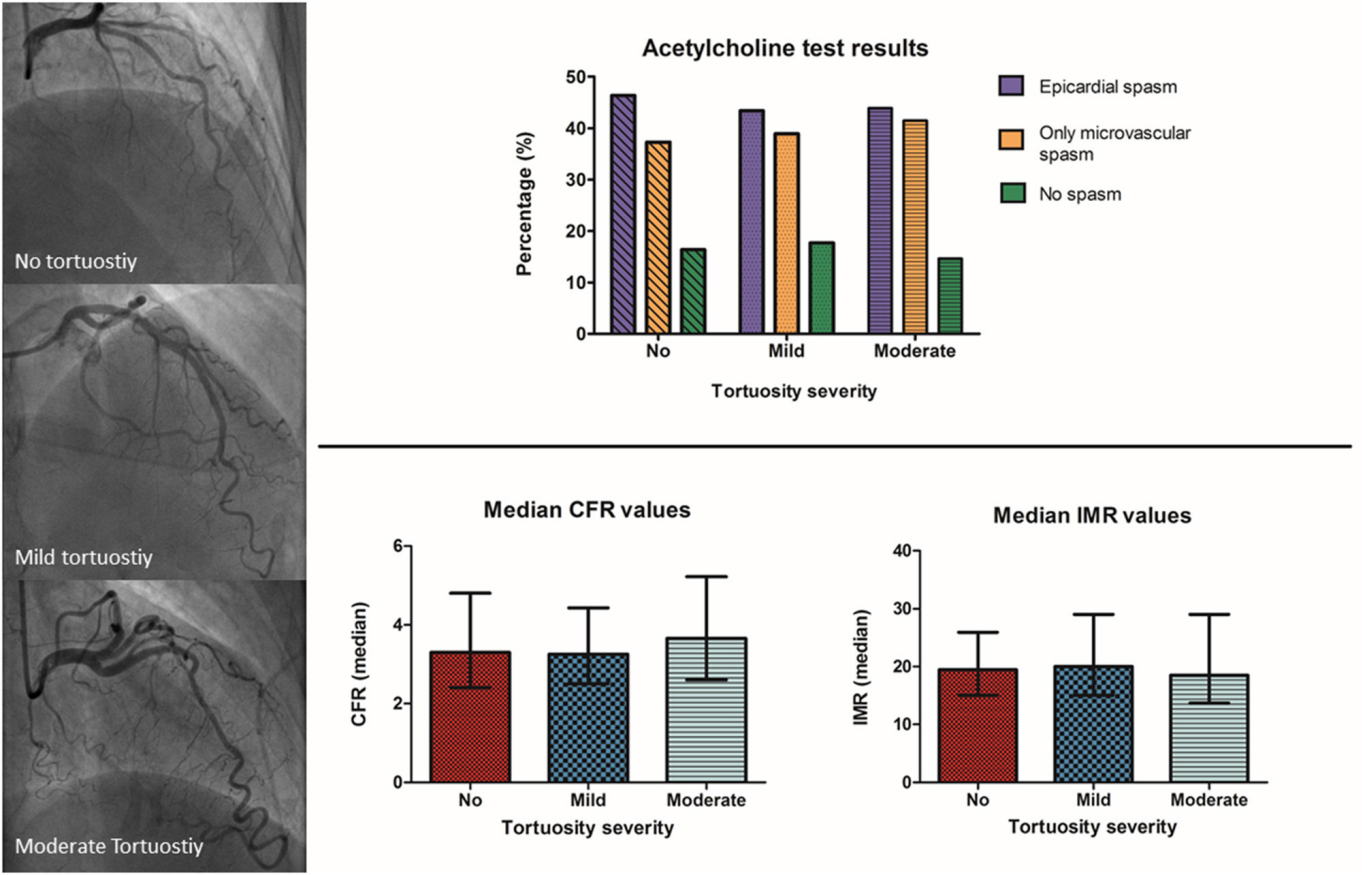
Title: Acetylcholine and adenosine testing results between the different severities of tortuosity. Top–Acetylcholine spasm test results showed no difference in the prevalence of epicardial and microvascular spasm between patients with angina and no obstructive coronary artery disease and moderate, mild or no coronary tortuosity. Bottom–Adenosine testing results showed no difference in the prevalence of microvascular dysfunction between patients with angina and no obstructive coronary artery disease and moderate, mild or no coronary tortuosity. ACH, acetylcholine; ADE, adenosine; CFR, coronary flow reserve; IMR, index of microvascular resistance.

Coronary spasm was present in 187 patients (81%), of which 87 patients (35%) had microvascular spasm and 100 patients (46%) had epicardial spasm. As displayed in Figure 3 and Table 3, we found no differences in the prevalence of both types of spasm between the no, mild and moderate tortuosity groups (46 vs. 43 vs. 44% of patients with epicardial spasm and 36 vs. 39 vs. 42% of patients with microvascular spasm, respectively, *p* = 0.98). Of the 100 patients with epicardial spasm, 33 patients (33%) had focal spasm and 76 patients (67%) had diffuse spasm. The prevalence of focal or diffuse epicardial spasm did not differ between the tortuosity groups. Correcting our analyses for gender and hypertension did not change our results (data not shown).

**Table 3:** Coronary function test and adenosine test results stratified for patients with no/mild/moderate tortuosity.

	**Total** ***N* = 228**	**No tortuosity** ***n* = 73**	**Mild tortuosity** ***n* = 114**	**Moderate tortuosity** ***n* = 41**	***p*-value**
**Acetylcholine testing**					0.98
Negative	39 (18%)	13 (18%)	20 (18%)	6 (15%)	
Microvascular spasm	87 (38%)	26 (36%)	44 (39%)	17 (42%)	
Epicardial spasm	100 (44%)	33 (46%)	49 (43%)	18 (44%)	
					0.26
Focal epicardial spasm	33 (33%)	8 (24%)	20 (41%)	5 (28%)	
Diffuse epicardial spasm	67 (67%)	25 (76%)	29 (59%)	13 (72%)	
**Adenosine measurements**					
Rest Tmn, sec	0.91 [0.56–1.26]	0.92 [0.57–1.27]	0.86 [0.51–1.21]	0.97 [0.67–1.27]	0.99
Hyperemic Tmn, sec	0.26 [0.16–0.35]	0.24 [0.17–0.31]	0.26 [0.17–0.35]	0.22 [0.11–0.33]	0.21
CFR	3.3 [2.2–4.4]	3.6 [2.2–5.0]	3.2 [2.2–4.3]	3.7 [2.4–5.0]	0.24
CFR <2.0	35 (16%)	11 (17%)	18 (16%)	6 (16%)	0.99
IMR	19.3 [13.3–25.3]	18.5 [13.8–23.2]	20.0 [13.0–27.0]	18.5 [10.7–26.3]	0.26
IMR ≥ 25	65 (30%)	15 (23%)	38 (35%)	12 (32%)	0.25

*Values are % (n) or median (interquartile interval)*.

*Tmn, mean transit time; CFR, coronary flow reserve; IMR, index of microvascular resistance*.

In an additional analysis, we evaluated the differences in coronary spasm prevalence between all patients with tortuosity vs. no tortuosity. No differences were found between the groups, as displayed in Supplementary Table 1.

### Microvascular Dysfunction and Tortuosity

In total 33 patients (15%) had an impaired CFR and 65 patients (30%) had an impaired IMR. As displayed in Table 3, we found similar CFR values (median = 3.4 [2.3–4.6]) and IMR values (median = 19.0 [13.0–25.0]) between the tortuosity groups. Also, the mean transit times at rest and hyperaemia did not differ between the groups. The prevalence of both an impaired CFR (no tortuosity, mild tortuosity, moderate tortuosity; 17, 16, and 16, respectively, *p* = 0.99) and an impaired IMR (no tortuosity, mild tortuosity, moderate tortuosity; 23, 35, and 32%, respectively, *p* = 0.25) did not differ between the tortuosity groups. In addition, there were no differences in the prevalence of MVD, comprising impaired CFR and/or increased IMR, between the tortuosity groups (no, mild, moderate; 36, 42, and 42%, respectively, *p* = 0.70). A multifactorial regression model, correcting for gender and a history of hypertension did not change these findings (CFR: *R* = 0.119, *p* = 0.39; IMR: *R* = 0.131, *p* = 0.30).

We extended our tortuosity analyses by comparing a combined group of mild tortuosity and moderate tortuosity with no tortuosity. This did not influence our findings (data not shown).

### Additional Markers of Tortuosity

Analyzing the additional markers of tortuosity, we found 6 patients (2.6%) with an intravessel symmetry sign, 5 patients (2.2%) with a corkscrew sign, and 50 patients (22%) with an S-curve sign. Because of the low prevalences, we only explored the role of the S-curve sign, as presented in Table 4. There were no significant differences in the prevalence of coronary spasm and MVD between patients with and without an S-curve sign.

**Table 4:** Coronary function test and adenosine test results stratified for patients with and without an S-curve sign.

	**No S-curve sign** ***n* = 178**	**S-curve sign** ***n* = 50**	***P*-value**
**Acetylcholine testing**			0.78
Epicardial spasm	77 (44%)	23 (46%)	
Microvascular spasm	67 (38%)	20 (40%)	
Negative	32 (18%)	7 (14%)	
**Adenosine measurements**			
CFR	3.5 [2.3–4.7]	3.3 [2.4–4.3]	0.42
CFR <2.0	26 (16%)	9 (19%)	0.62
IMR	19.0 [13.5–24.5]	21.5 [15.0–28.0]	0.30
IMR ≥ 25	46 (28%)	19 (40%)	0.12

*Values are % (n) or median (interquartile interval)*.

*Abbreviations as in Table 3*.

To evaluate the influence of the amount of curvatures on flow, we also assessed the number of curvatures for each LAD. We found a median of 4 curvatures per LAD, ranging from 0 to 10 curvatures per patient. There was no correlation between the number of curvatures and CFR (*r* = −0.77, *p* = 0.27), but a trend toward correlation with IMR (*r* = 0.124; 95% CI: −0.009 to 0.253; *p* = 0.07). However, the number of curvatures did correlate with IMR when corrected for gender and hypertension (*r* = 0.149; *p* = 0.03).

## Discussion

To our knowledge, this is the first study to investigate the association between the different endotypes of CVDys—as assessed by CFT—and coronary tortuosity, in patients with ANOCA. Including an analysis of 228 patients, we found no association between coronary tortuosity and the different endotypes of coronary vasomotor dysfunction, including both coronary spasm and MVD.

### Coronary Tortuosity and Vasospasm

We did not find any association between tortuosity and vasospasm. Based on previous studies, we hypothesized that tortuosity and vasospasm, as an expression of endothelial dysfunction, might be related through WSS. Kumar et al. (8), previously evaluated the relationship between endothelial dysfunction and WSS in 44 patients with ANOCA and showed that low WSS was independently associated with severe endothelial dysfunction, defined as >10% vasoconstriction after acetylcholine (Ach) infusion. They hypothesized that low WSS leads to loss of endothelial nitric oxide synthase and decreased nitric oxide production, which in turn leads to impairment of normal dilatory response of coronary arteries and ultimately vasospasm (8). Low WSS was especially situated in the inner curvatures of the vessels. In this way, increased tortuosity, characterized by a high number of curvatures in the coronary arteries, might be associated with vasospasm via low WSS. In contrast, Li et al. (9) demonstrated that tortuosity was associated with higher WSS, but this was not a clinical study but an *ex-vivo* experiment (9).

### Coronary Tortuosity and Microvascular Dysfunction

In our study, we did not find differences in microvascular function indices (IMR and CFR) between the tortuosity groups. It has been hypothesized that increased tortuosity can lead to flow alteration, resulting in a diminished coronary pressure distal to the tortuous segment of the coronary artery, possibly leading to ischemia (6). Our findings do not confirm this hypothesis. Although we used the same definitions of tortuosity and measurements of MVD, we also could not replicate the results of the small pilot study by Li et al. (11) who did find a lower CFR and higher IMR in 8 patients with tortuosity compared to patients without tortuosity. Future studies are warranted to further explore the causality between flow alterations caused by coronary tortuosity and ischemia.

### Similar Etiologies of Coronary Tortuosity and CVDys?

Our finding that women more often develop coronary tortuosity than men is in agreement with various previous studies (17–21). Women also tend to have a higher prevalence of CVDys than men (22), suggesting that coronary tortuosity and CVDys might have similar etiologies. Some studies have linked tortuosity with smaller heart size (18, 23), which may possibly also explain why women have more coronary tortuosity than men (18, 24, 25).

Although not statistically significant, hypertension tended to be more prevalent with increasing severity of tortuosity. Hypertension was also positively associated with coronary tortuosity in the study by Li et al. (17) and has also been related to CVDys (1). In this way, hypertension may be a cause of both coronary tortuosity and CVDys. Furthermore, we found that patients with angina exerted by emotions or stress, which could trigger hypertension, have a higher prevalence of tortuosity. Larger studies are needed to address this issue in depth.

### Study Limitations

First, since our center is a tertiary care hospital specialized in CVDys, we found a high prevalence of CVDys (83%) in our cohort. Also, the prevalence of epicardial spasm (46%) and microvascular spasm (35%) appears to be higher than that of previous studies (~33 and ~24%) (26, 27). This limits our ability to compare patients with CVDys and those without CVDys. On the other hand, the high prevalence of CVDys in our cohort did enable us to compare the various endotypes of CVDys to the amount of coronary tortuosity. Second, not all patients who underwent coronary function testing completed the adenosine tests for MVD, which may have caused information bias. However, we could still evaluate the relation between coronary artery spasm and tortuosity in these patients.

Third, there is currently no standardized, universally accepted method for measuring and reporting coronary tortuosity in literature. We chose to apply the classification of tortuosity set up by Eleid et al. (7). However, according to this classification there were no patients with severe tortuosity, defined as the presence of ≥2 consecutive curvatures of ≥180° in the LAD artery, in our cohort of 228 patients. Although this implied that we could not evaluate the severe tortuosity group, Eleid et al. (7) found a similar rate of severe tortuosity patients in non-obstructive CAD, namely 1 patient out of 313 patients. They found, however, more severe tortuosity in patients with a spontaneous coronary artery dissection, suggesting the classification is clinically relevant and usable in other patient categories. Also, reference values for the number of curvatures in the LAD artery have not been clinically validated yet like the cut-offs for the CFR and IMR. We were the first study that compared patients with high number of curvatures to those with low number of curvatures based on the median of curvatures. Hence, this cut-off should be optimized in future trials with adequate power. Furthermore, we were not able to perform additional marker analyses on the intravessel symmetry sign and corkscrew sign, because these signs were only recognized in a few patients in this study cohort.

Fourth, we cannot conclude that there is no association between CVDys and coronary tortuosity in the circumflex artery or right coronary artery, because measurements were only performed in the LAD artery. However, we do not have reason to believe that the relation would be different in these coronary arteries. We performed the CFT in line with the current guidelines (5, 28) in which the LAD artery is examined as it provides a representative picture of CVDys.

Last, as the majority of our patients were female it would have been of interest to know the prevalence of fibromuscular dysplasia (FMD), which is a another strong determinant of arterial tortuosity dominating in women (16). This will be subject of further studies.

## Conclusion

In 228 patients with ANOCA undergoing a clinically indicated CFT, we did not find an association between coronary tortuosity and the various endotypes of coronary vasomotor dysfunction. Future experimental and clinical studies on the complex interplay between coronary tortuosity, wall shear stress, endothelial dysfunction, and coronary flow are warranted.

## Data Availability Statement

The raw data supporting the conclusions of this article will be made available by the authors, without undue reservation.

## Ethics Statement

The studies involving human participants were reviewed and approved by CCMO Arnhem Nijmegen. The patients/participants provided their written informed consent to participate in this study.

## Author Contributions

TJ, KK, NR, PD, and SE-S contributed to conception and design of the study. TJ and KK organized the database and performed the statistical analysis. KK wrote the first draft of the manuscript. TJ, SE-S, and AM wrote sections of the manuscript. All authors contributed to manuscript revision, read, and approved the submitted version.

## Conflict of Interest

The authors declare that the research was conducted in the absence of any commercial or financial relationships that could be construed as a potential conflict of interest.

## Publisher's Note

All claims expressed in this article are solely those of the authors and do not necessarily represent those of their affiliated organizations, or those of the publisher, the editors and the reviewers. Any product that may be evaluated in this article, or claim that may be made by its manufacturer, is not guaranteed or endorsed by the publisher.
