# Supplementary material for: Relation Between Coronary Tortuosity and Vasomotor Dysfunction in Patients Without Obstructed Coronaries?
Source: Front Cardiovasc Med. 2022 Jan 13;8:804731. doi: 10.3389/fcvm.2021.804731 (PMC8792852; doi:10.3389/fcvm.2021.804731)
Supplement: Supplementary file 1 [file Table_1.docx]

|  | **No tortuosity**  **N=73** | **Tortuosity**  **N=155** | **p-value** |
| --- | --- | --- | --- |
| **Acetylcholine testing** |  |  | 0.88 |
| Epicardial spasm | 33 (46%) | 67 (43%) |  |
| Microvascular spasm | 26 (36%) | 61 (40%) |  |
| Negative | 13 (18%) | 26 (17%) |  |
| **Adenosine measurements** |  |  |  |
| CFR | 3.5 [2.1 – 4.9] | 3.4 [2.3 – 4.4] | 0.26 |
| CFR < 2.0 | 15 (10) | 13 (20) | 0.83 |
| IMR | 19.0 [14.2 – 23.9] | 19.0 [12.0 – 26.0] | 0.23 |
| IMR ≥ 25 | 15 (23%) | 50 (34%) | 0.10 |
| **Angina characteristics** | | | |
| Symptoms at rest | 52 (92%) | 107 (86%) | 0.29 |
| Symptoms during exercise | 46 (81%) | 95 (76%) | 0.48 |
| Symptoms exerted by emotion or stress | 32 (56%) | 89 (71%) | 0.05 |

Table S1. Acetylcholine test and adenosine test results stratified for patients without/with tortuosity.

Values are % (n) or median (interquartile interval).
CFR = coronary flow reserve; IMR = index of microvascular resistance.
